# Association between transfusion status and clinical and economic outcomes in patients with myelodysplastic syndromes from the physicians' perspective

**DOI:** 10.1002/cnr2.1680

**Published:** 2022-08-09

**Authors:** Shaloo Gupta, Austin G. Kulasekararaj, Halley Costantino, Jay Grisolano, Jackson Tang, Shalon Jones, Derek Tang

**Affiliations:** ^1^ Cerner Enviza Malvern Pennsylvania USA; ^2^ NIHR Wellcome King's Clinical Research Facility, King's College Hospital NHS Foundation Trust London and King's College London London UK; ^3^ Bristol Myers Squibb Princeton New Jersey USA

**Keywords:** blood transfusion, hospital costs, myelodysplastic syndromes, physicians, survey, treatment outcome

## Abstract

**Background:**

The current study investigated physicians' understanding of the impact of transfusion status (TS) on clinical and economic outcomes in patients with myelodysplastic syndromes (MDS).

**Materials & Methods:**

378 physicians primarily specializing in hematology/oncology across five European countries completed the survey. The survey asked physicians for their perspectives on the impact of TS on risk of death, risk of progression to acute myeloid leukemia (AML), chance of leukemia‐free survival, and number of significant bleeding events, infection events, hospitalizations, and emergency room (ER) visits.

**Results:**

Physicians estimated that compared to transfusion‐dependent (TD) patients, transfusion‐independent (TI) patients had a 37.6% reduced risk of death, lower risk of progression to AML, and lower risk of non‐leukemic death, for all MDS risk levels. TD patients who became TI after treatment were estimated to have 40.6% reduced risk of death and 34% reduced risk of progression to AML, compared to TD patients who remained TD.

**Conclusions:**

Compared with TD patients, physicians estimated that TI patients have fewer events of infection and significant bleeding, and experience fewer hospitalizations and ER visits per person per year. Overall, physicians reported better outcomes for TI patients. New treatment options for patients with MDS to reduce or eliminate transfusion burden are warranted.

## 1 INTRODUCTION

Myelodysplastic syndromes (MDS) are a diverse group of clonal hematopoietic stem cell malignancies characterized by bone marrow failure, peripheral blood cytopenias, and reduced survival.^1^, ^2^ Patients with MDS experience a high symptom burden resulting in decreased quality of life.^3^ Supportive care (including transfusions, growth factors, and antibiotic treatment) forms the basis for most MDS therapy plans and helps to reduce symptoms and the risk of disease progression and death.^4^

The MDS population shows a wide variation in their risk of death or leukemia progression and a number of risk stratification systems have been developed to more accurately estimate outcomes and aid clinical decision making. The International Prognostic Scoring System (IPSS) was developed based on data of patients with untreated and primary MDS.^5^ It stratifies patients into four risk groups, using information on cytogenic status, number of cytopenias, and bone marrow blast percentage. The risk groups are designated as Low, Intermediate‐1, Intermediate‐2, and High risk. Due to limitations of this system, a revised IPSS (IPSS‐R) was developed which includes the same disease factors but with more detailed information.^6^ Patients with higher‐risk disease are stratified into IPSS categories of Intermediate‐2 and High‐risk groups, broadly corresponding to the IPSS‐R groups Very high, High, and, sometimes, Intermediate. Based on the IPSS classification, higher‐risk patients (Intermediate‐2 and High‐risk groups) generally have a worse prognosis, with an estimated median overall survival (OS) of 0.4 years, in comparison to 5.7 years among patients with lower‐risk disease (Intermediate‐1 and Low‐risk groups).^7^ Additionally, higher‐risk patients are more likely to die from disease‐related causes such as bleeding, infections, and progression to acute myeloid leukemia (AML).^8^

The majority of patients with MDS have anemia due to the malignancy, and up to 90% of patients require red blood cell (RBC) transfusions during the course of their disease. Due to the chronic nature of MDS‐associated anemia, many patients develop long‐term dependence on RBC transfusions.^9^ In the lower‐risk group, 22% of patients are transfusion dependent (TD), in contrast to 68% for higher‐risk MDS.^10^ TD is associated with substantial clinical, health‐related quality of life (HRQoL), and economic consequences. TD patients have significantly more hospitalizations, infectious complications, and a shorter OS than transfusion independent (TI) patients.^11^, ^12^ The cost of repeated transfusions and more frequent hospitalizations contribute to a higher economic burden, with TD patients incurring double the medical costs of TI patients.^3^

Few studies have examined physicians' perspectives on the relationship between transfusion dependence and outcomes in MDS. Most existing literature has reported physicians' perspectives on survival outcomes; other clinical outcomes such as bleeding, infection, and economic outcomes such as healthcare resource utilization have not previously been examined. The current survey was performed to investigate physicians' understanding of the impact of transfusion status (TS) on the risk of death (i.e., OS), progression to AML, chance of leukemia‐free survival (LFS), risk of non‐leukemic death, bleeding and infection events, as well as hospitalizations and emergency room (ER) visits.

## 2 METHODS

### 2.1 Participants

Physicians based in the United Kingdom, France, Germany, Italy, or Spain were recruited through a healthcare professionals panel (M3) and were considered eligible for the survey if they were practicing medicine for between 2 and 35 years, specialized in hematology and/or oncology with at least 75% of their time spent on direct patient care, and had managed ≥15 patients with MDS in the past 3 months. It was mandatory for participants to provide informed consent before entering the survey.

Physicians received renumeration for their participation, the amount of which was determined according to the fair market value in each country for a research interview. The study protocol was approved with exemption status by Pearl Institutional Review Board (Indianapolis, IN, USA).

### 2.2 Survey design

The survey was performed in 2 phases and was provided to the physician in their native language. The survey was developed in English and was translated into local languages (French, Italian, German, and Spanish) by Global Lexicon (ISO 9001‐accredited) by trained native‐speaker linguists familiar with health outcomes research and medical terminology. Then the translated versions were back‐translated by Language Insight (ISO 9001‐accredited). In the first phase, cognitive interviews were conducted with a small subset of physicians (*N* = 3 in each country) to pre‐test and revise the questionnaire in January and February 2020. This ensured that the survey content was relevant and easy to comprehend.

The second phase consisted of a web‐based survey to generate quantitative data, which was fielded in April and May 2020. The survey assessed physician demographics and their perspectives on the impact of MDS risk group and TS on risk of death, AML progression, chance of LFS, significant bleeding events, number of infection events, hospitalizations, and ER visits. Physicians provided responses based on their own clinical experience, beliefs, and knowledge of the literature. In the instructions accompanying the survey, physicians were provided with a definition of transfusion dependence (≥1 unit[s] every 8 weeks), transfusion burden (high burden: ≥4 units every 4 weeks), and MDS IPSS and IPSS‐R risk levels to ensure consistency across all physicians. Physicians were asked to estimate outcomes between lower‐ and higher‐risk patients. The higher‐risk group was defined as IPSS‐R scores of Very high, High, or Intermediate‐2 and IPSS scores of High or Intermediate‐2. IPSS‐R scores of Very low, Low or Intermediate‐1 and IPSS scores of Low or Intermediate‐1 were defined as lower risk.

Participants were sent a unique survey link via email (including appropriate introduction to the research and an informed consent form) after qualifying for either the first or second phase. The consent form informed potential respondents that participation was voluntary and that responses would remain confidential. It also provided information about the study aims, the estimated survey duration, and incentives for participation. Lastly, the statement of informed consent provided potential respondents with the resources to address any concerns they may have. After reading the statement of informed consent, the respondents had to select the option “I agree to participate” to enter the survey. For those who selected “I do not agree to participate,” study participation was terminated. Following completion of the first phase, participants were paid from $87.80 to $170.00 (USD) in the equivalent local currency. After completion of the second phase, participants were paid the equivalent of $102.00 (USD) in France, Germany, Italy, and the United Kingdom, and $92.00 (USD) in Spain. An abridged version of the online survey is available in the supporting material.

Physicians were not aware of the study sponsor and the sponsor was not given any identifying information on the participating physicians. Physicians in France were informed that Bristol Myers Squibb was the sponsor due to General Data Protection Regulation requirements. However, this information was provided at the end of the survey when it was no longer possible for the physicians to modify responses, thereby ensuring that their responses were not influenced by this information.

### 2.3 Statistical analysis

Results are described descriptively in proportions for categorical/ordinal data and mean with standard error (SE) or 95% confidence interval (CI) for continuous data. Overall analysis of variance (ANOVA) tests to compare across countries were conducted (alpha level = 0.05). Scheffé's multiple comparison adjustments were also conducted for outcomes found significant with the overall ANOVA test.

## 3 RESULTS

### 3.1 Physician study population

**FIGURE 1:**
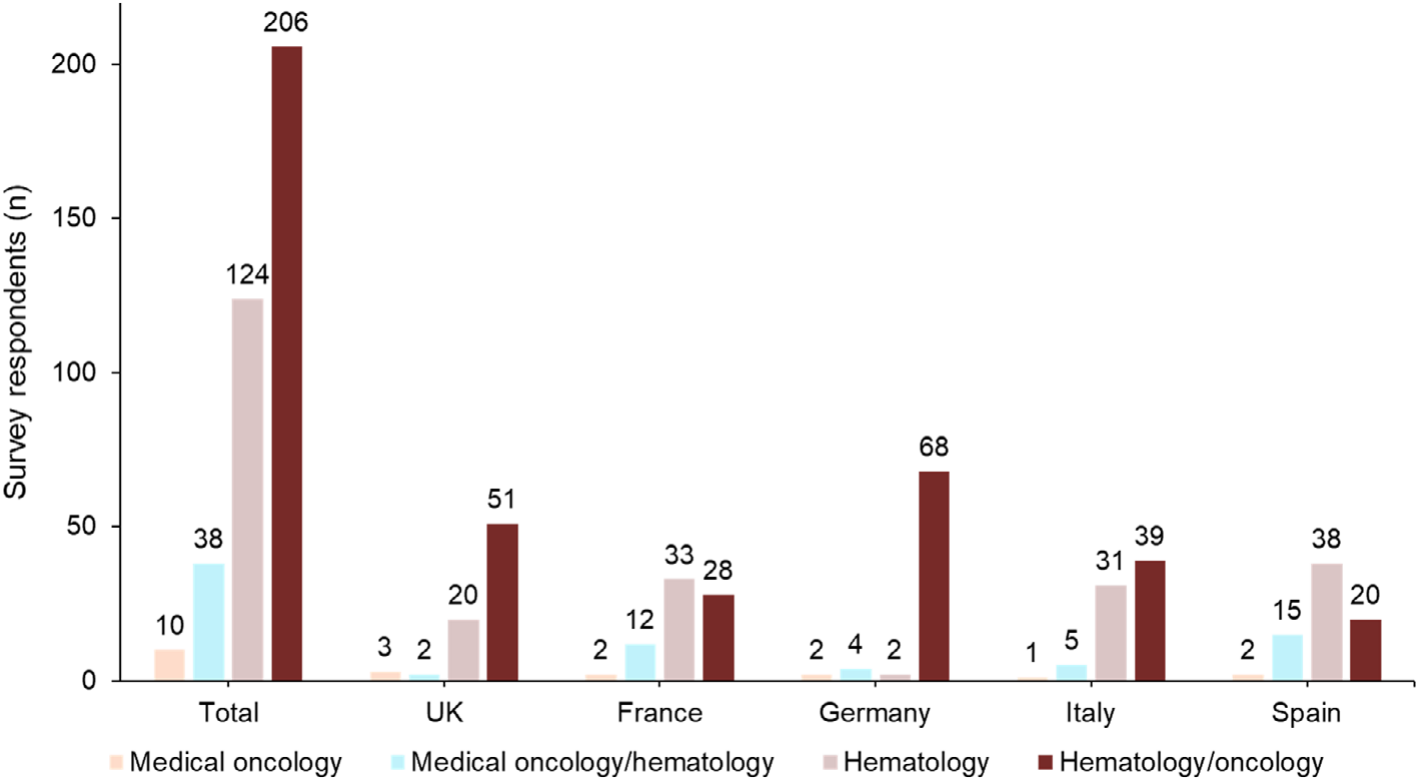
Physician specialties overall and by country

In total, 378 physicians from the participating countries completed the survey (*n* ≈ 75 in each country); a total of 244 hematologist/oncologists, 124 hematologists, and 10 oncologists participated (Figure 1). Physician demographics are presented in Table 1 (data also under review in another manuscript). The majority of participants were male (64.3%), with only Italy reporting a majority of female physicians (53.9%). Most physicians were aged between 45 and 54 years (41.3%) or 35 to 44 years (36.5%), with Italy reporting the highest percentage of physicians aged under 35 years (23.7%) and Germany reporting the highest percent of physicians aged 55 to 64 years (21.1%). Only 1 physician in France was aged 65 years or older.

**TABLE 1:** Physician characteristics by country

Characteristic	Total *N* = 378	UK *n* = 76	France *n* = 75	Germany *n* = 76	Italy *n* = 76	Spain *n* = 75
Age, *n* (%), years						
<35	28 (7.4)	5 (6.6)	3 (4.0)	1 (1.3)	18 (23.7)	1 (1.3)
35–44	138 (36.5)	32 (42.1)	23 (30.7)	24 (31.6)	26 (34.2)	33 (44.0)
45–54	156 (41.3)	33 (43.4)	35 (46.7)	35 (46.1)	25 (32.9)	28 (37.3)
55–64	55 (14.6)	6 (7.9)	13 (17.3)	16 (21.1)	7 (9.2)	13 (17.3)
≥65	1 (0.3)	0 (0.0)	1 (1.3)	0 (0.0)	0 (0.0)	0 (0.0)
Gender, *n* (%)						
Male	243 (64.3)	49 (64.5)	54 (72.0)	63 (82.9)	35 (46.1)	42 (56.0)
Female	135 (35.7)	27 (35.5)	21 (28.0)	13 (17.1)	41 (53.9)	33 (44.0)
Years in practice, mean (SE)	14.70 (0.32)	13.05 (0.59)	16.75 (0.70)	14.46 (0.64)	13.29 (0.69)	16.00 (0.75)
% Professional time spent in direct patient care, mean (SE)	88.72 (0.36)	87.70 (0.73)	89.64 (0.80)	88.28 (0.78)	89.66 (0.87)	88.35 (0.83)
No. of MDS patients seen and/or treated in past 3 months, mean (SE)	54.47 (2.26)	41.53 (3.71)	59.68 (5.43)	55.97 (5.26)	63.14 (5.10)	52.08 (5.38)
MDS classification categories used by physicians, *n* (%)						
IPSS	158 (41.8)	39 (51.3)	40 (53.3)	30 (39.5)	32 (42.1)	17 (22.7)
IPSS‐R	220 (58.2)	37 (48.7)	35 (46.7)	46 (60.5)	44 (57.9)	58 (77.3)
% TD patients, mean (SE)	47.41 (1.02)	46.84 (2.35)	45.04 (2.18)	50.67 (2.55)	47.95 (2.20)	46.52 (2.06)
Practice locations, *n* (%)						
Urban	334 (88.4)	56 (73.7)	69 (92.0)	68 (89.5)	72 (94.7)	69 (92.0)
Suburban	39 (10.3)	20 (26.3)	6 (8.0)	5 (6.6)	4 (5.3)	4 (5.3)
Rural	5 (1.3)	0 (0.0)	0 (0.0)	3 (3.9)	0 (0.0)	2 (2.7)
No. of oncologists/hematologists in the practice, *n* (%)						
1	6 (1.6)	0 (0.0)	0 (0.0)	6 (7.9)	0 (0.0)	0 (0.0)
2–4	107 (28.3)	20 (26.3)	18 (24.0)	46 (60.5)	13 (17.1)	10 (13.3)
5–10	116 (30.7)	26 (34.2)	30 (40.0)	12 (15.8)	22 (28.9)	26 (34.7)
>10	149 (39.4)	30 (39.5)	27 (36.0)	12 (15.8)	41 (53.9)	39 (52.0)

Abbreviations: IPSS‐R, revised International Prognostic Scoring System; MDS, myelodysplastic syndromes; SE, standard error; TD, transfusion dependent.

On average, physicians had been practicing medicine for 14.7 years and dedicated 88.7% of their professional time to direct patient care. Physicians estimated that they had treated an average of 54.5 patients with MDS in the past 3 months and that nearly half (47.4%) of their patients were TD. A lower percentage of physicians use the IPSS (41.8%) in comparison to the newer IPSS‐R (58.2%) (Table 1).

Across participating countries, the majority of practices were urban (≥73.7%). The United Kingdom was the only country to report a considerable proportion of suburban practices (26.3%). Rural practices were only reported in Germany (3.9%) and Spain (2.7%). Overall, 39.4% of physicians were working in large practices with >10 oncologists/hematologists. However, 60.5% of physicians in Germany were working in relatively smaller practices with 2–4 oncologists/hematologists (Table 1).

### 3.2 Risk of death

**FIGURE 2:**
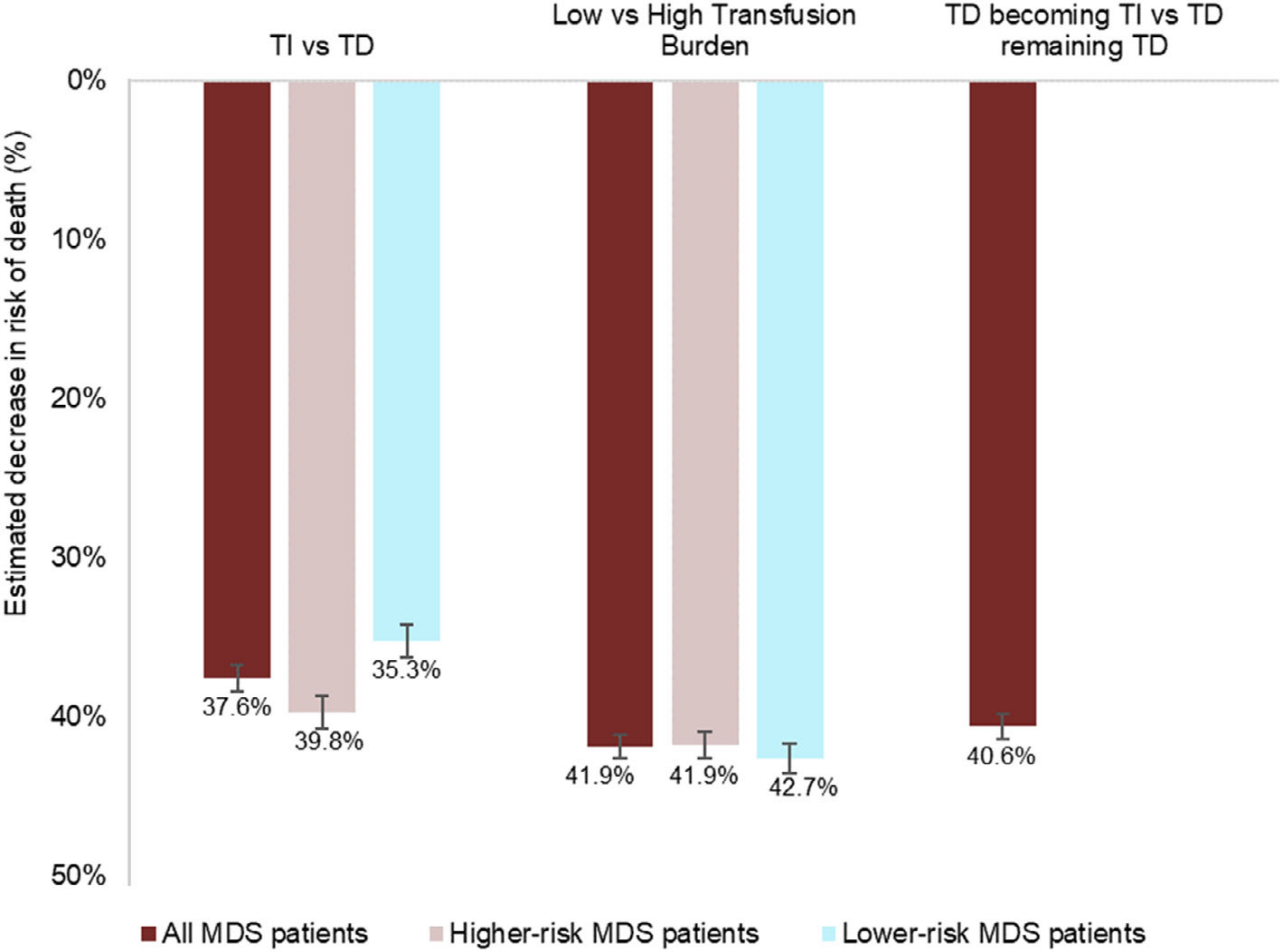
Estimated decreases in risk of death across different TS groups in patients with MDS. Numbers below the bars are the mean; error bars are SE. MDS, myelodysplastic syndromes; SE, standard error; TD, transfusion dependent; TI, transfusion independent; TS, transfusion status.

Physician‐reported estimates of decrease in risk of death between (i) TD and TI patients; (ii) low and high transfusion burden patients (stratified by risk group); and (iii) between TD patients that become TI against TD patients that remain TD (across all risk groups) are shown in Figure 2. Physicians estimated that in comparison to TD patients, TI patients had a reduced risk of death of 37.6% (SE = 0.87; 95% CI: 35.9%, 39.3%) for all risk levels. TI in higher‐risk patients was reported to have a greater reduction in risk of death (39.8%; SE = 1.03; 95% CI: 37.7%, 41.8%) than in lower‐risk patients (35.3%; SE = 1.06; 95% CI: 33.2%, 37.4%). Physicians estimated that patients with a low transfusion burden would have an even greater reduction in risk of death of 41.9% (SE = 0.75; 95% CI: 40.4%, 43.4%) across all risk levels, with similar estimates for low‐ and high‐risk levels. TD patients who became TI after treatment were estimated to have a risk reduction of 40.6% (SE = 0.79; 95% CI: 39.1%, 42.2%) in comparison to TD patients who remained TD after treatment.

**FIGURE 3:**
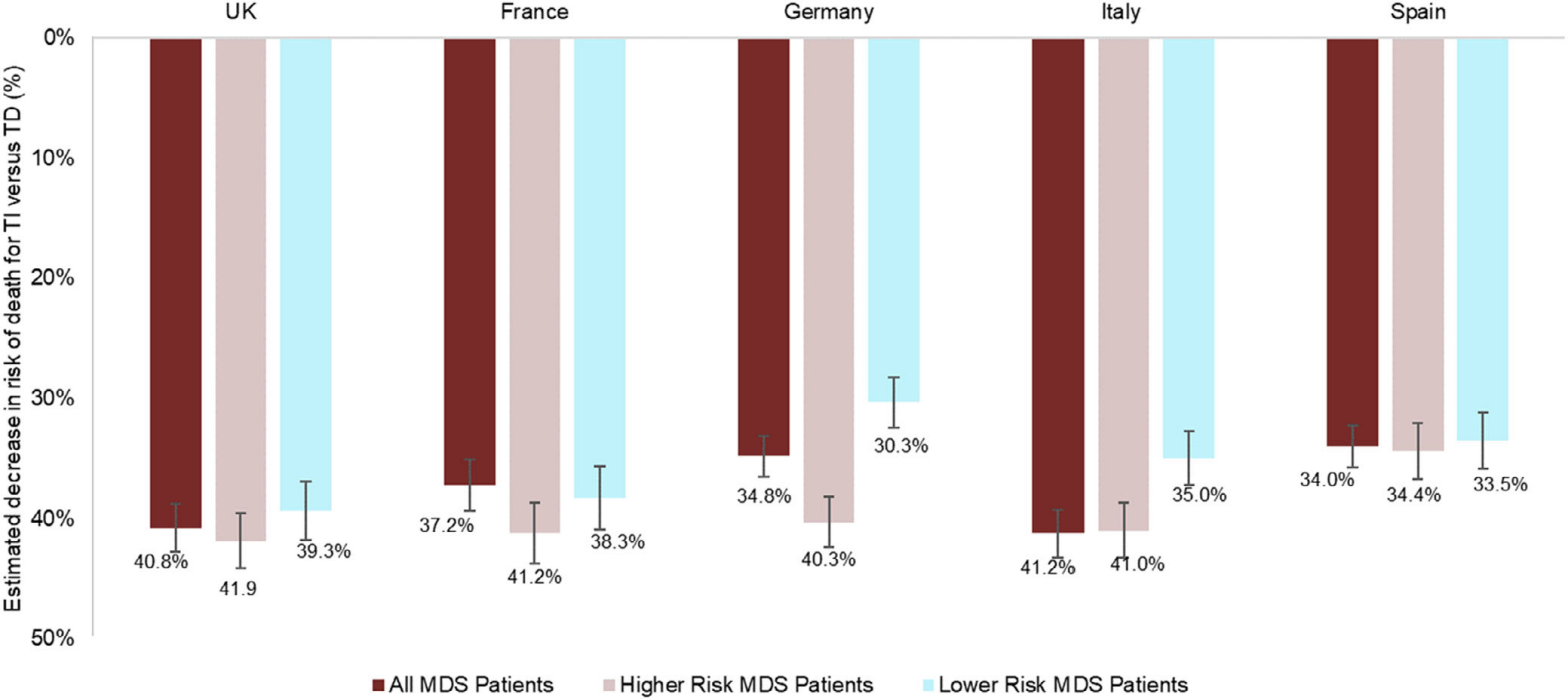
Estimated decreases in risk of death between TI and TD patients, overall and for higher and lower risk groups by country. Numbers below the bars are the mean; error bars are SE. MDS, myelodysplastic syndromes; SE, standard error; TD, transfusion dependent; TI, transfusion independent.

Physician‐reported estimates of decrease in risk of death, overall and for higher and lower risk groups specifically, between TI and TD patients are shown by country in Figure 3. When comparing estimates between countries across all categories and levels, no strong trends were observed. The only significant difference between countries was for reduction in risk of death in Low versus High transfusion burden for lower risk level patients, with physicians in the UK (45.9% vs. 36.4%; *p* = .037) and France (46.0% vs. 36.4%; *p* = .033) estimating a greater reduction in risk of death in lower‐risk patients than physicians in Germany.

### 3.3 Risk of progression to AML

Among all patients with MDS, physicians estimated that TI patients have a 38.4% (SE = 0.89; 95% CI: 36.7%, 40.2%) lower risk of progression to AML compared with TD patients, ranging from 34.7% (SE = 1.85; 95% CI: 31.0%, 38.4%) in Germany to 41.0% (SE = 2.21; 95% CI: 36.6%, 45.5%) in France. Physicians estimated that TD patients who became TI after treatment would have a risk of progression to AML or death that was reduced by 34.5% (SE = 0.84; 95% CI: 32.8%, 36.1%) in lower‐risk MDS and by 33.5% (SE = 0.77; 95% CI: 32.0%, 35.0%) in higher‐risk MDS, compared with TD patients who remained TD after treatment. For both lower‐ and higher‐risk MDS, physicians in the United Kingdom reported the greatest relative reductions while physicians in Spain reported the lowest estimates.

### 3.4 Risk of non‐leukemic death and chance of LFS

Compared with TD patients, physicians estimated that TI patients have a lower risk of non‐leukemic death of 37.4% (SE = 0.79; 95% CI: 35.8%, 38.9%) for all risk levels, with a similar estimate for lower‐risk MDS and a lower estimate of 35.3% (SE = 0.82; 95% CI: 33.7%, 36.9%) for higher‐risk MDS. A significant difference between countries for TI versus TD regarding the reduction of risk of non‐leukemic death in lower‐risk MDS was found, with physicians in the United Kingdom (*p* < .001) and France (*p* = .015) estimating a greater reduction than physicians in Germany. Compared with TD patients who became TI after treatment, physicians estimated that TD patients who remained TD after treatment would have a 40.0% (SE = 0.83; 95% CI: 38.4%, 41.7%) decreased chance of LFS across all MDS risk groups. When stratified by lower‐ and higher‐risk MDS groups, the estimates were slightly lower at 37.5% (SE = 0.91; 95% CI: 35.7%, 39.3%) and 36.8% (SE = 0.97; 95% CI: 34.9%, 38.8%), respectively. For the higher‐risk MDS group, physicians in Spain reported the lowest reductions for both non‐leukemic death and chance of LFS, while physicians in the United Kingdom reported the greatest reductions. For the lower‐risk MDS group, physicians in Germany reported the lowest reductions for both outcomes. These estimates were significantly lower than the greatest reductions reported by physicians in France (*p* = .038) for chance of LFS.

### 3.5 Other clinical outcomes

**FIGURE 4:**
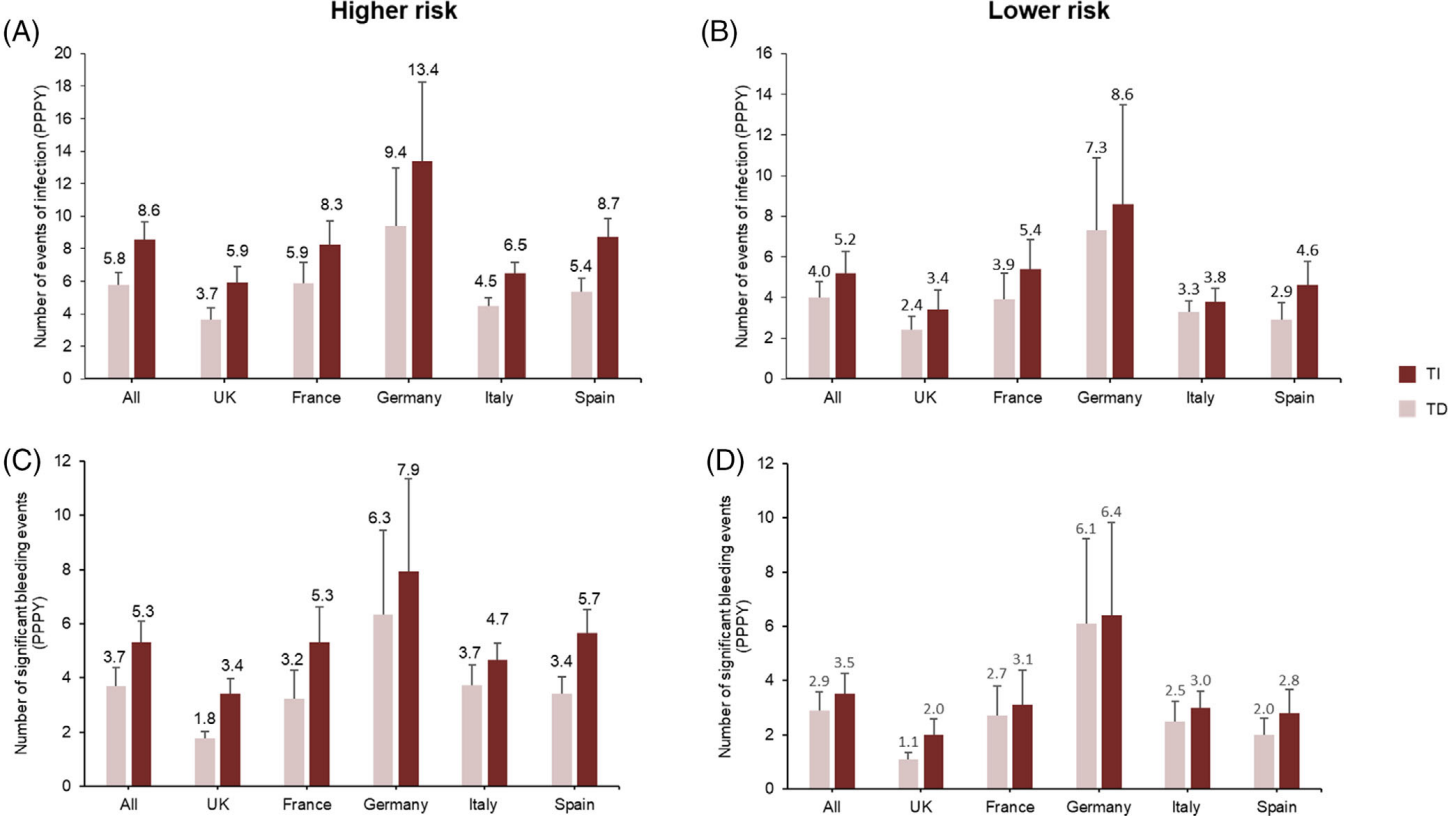
Estimated number of infection events (A and B) and significant bleeding events (C and D) in TI and TD patients with higher risk (A and C) and lower risk MDS (B and D) by country. Numbers above the bars are the mean; error bars are SE. MDS, myelodysplastic syndromes; PPPY, per person per year; SE, standard error; TD, transfusion dependent; TI, transfusion independent.

Physician‐reported estimates of infection rates in TI and TD patients are shown in Figure 4A,B. Compared with TD patients, physicians estimated that TI patients have fewer events of infection (per person per year [PPPY]): 2.8 (SE = 1.34) fewer among patients with higher‐risk MDS and 1.2 (SE = 1.23) fewer among those with lower‐risk MDS. Among both lower‐ and higher‐risk MDS groups, physicians in Italy reported the smallest difference in estimated infection rate between TD and TI patients. Physicians in Germany and Spain reported the greatest difference in infection rate between TD and TI patients for higher‐ and lower‐risk MDS, respectively.

Physician‐reported estimates of significant bleeding events in TI and TD patients are shown in Figure 4C,D. Among patients with higher‐risk MDS, physicians estimated that TI patients have 1.7 (SE = 1.04) fewer significant bleeding events PPPY than TD patients. Physicians in Spain reported the greatest difference between TI and TD patients, while physicians in Italy reported the smallest difference. Physicians estimated minimal differences in the rate of significant bleeding events between TI and TD patients for lower‐risk MDS.

### 3.6 Economic outcomes

**FIGURE 5:**
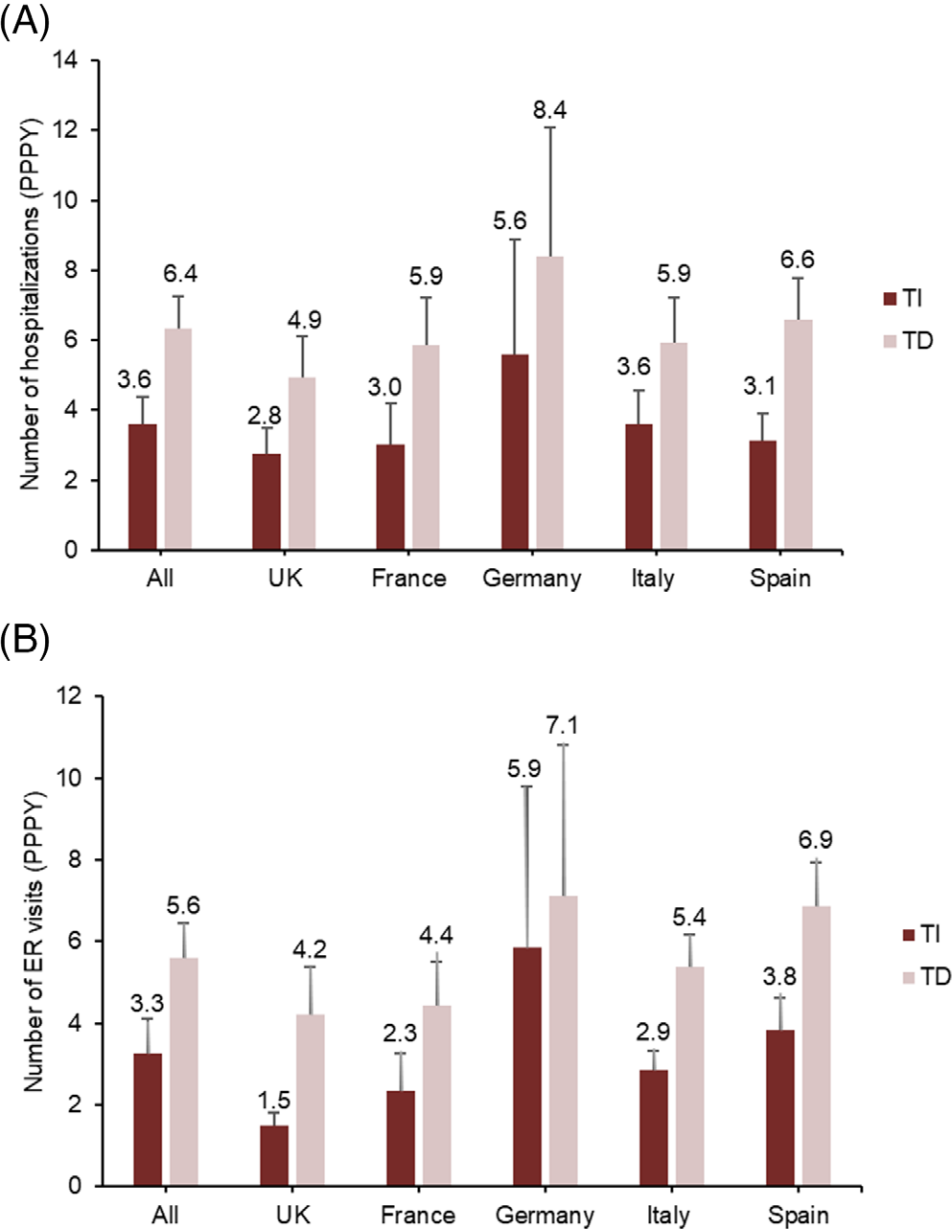
Estimated number of (A) hospitalizations and (B) ER visits for TI and TD patients by country. Numbers above the bars are the mean; error bars are SE. ER, emergency room; MDS, myelodysplastic syndromes; PPPY, per person per year; SE, standard error; TD, transfusion dependent; TI, transfusion independent.

The estimated number of hospitalizations and ER visits for TI and TD patients are shown in Figure 5A,B, respectively. Compared with TD patients, physicians estimated that TI patients have 2.8 (SE = 1.16) fewer hospitalization events and 2.3 (SE = 1.19) fewer ER visits PPPY. Physicians in Spain reported the greatest reduction in both hospitalization events and ER visits.

## 4 DISCUSSION

The current study surveyed physicians from five European countries to examine their perspectives on the impact of TS on disease outcomes for higher and lower MDS risk groups. Overall, physicians reported better outcomes for TI patients, with similar results reported across patients with different risk levels and across the different countries surveyed.

Multiple studies have shown that transfusion independence in MDS has a positive impact on OS. A meta‐analysis investigating the association between TI and OS in patients with MDS estimated a 59% reduction in mortality among TI patients when compared with TD patients.^9^ In the present study of physicians' perceptions, participating physicians estimated that TI patients had a 38% lower overall risk of death than TD patients. Although this estimate is lower than that obtained from the aforementioned meta‐analysis,^9^ it nevertheless highlights that the physicians surveyed recognize the benefit of transfusion independence in MDS. Physicians also estimated that if TD patients became TI after treatment, this would reduce the risk of death by 41% compared with TD patients who remained TD after treatment. This estimate is in line with the results of a phase 3 study of lenalidomide in patients with Low‐/intermediate‐ risk MDS with a 5q deletion, which showed a 47% reduction in the relative risk of death in TD patients who were TI for ≥8 weeks.^13^

Approximately 30% of patients with MDS eventually progress to AML. In this study, physicians estimated a lower risk of progression to AML for TD patients that become TI after treatment. Correspondingly, physicians reported that TD patients who remained TD after treatment would have a lower chance of LFS. In a cohort of lower‐risk patients, TD has been shown to be significantly associated with an increased risk of progression to AML.^14^ Studies of lenalidomide treatment in patients with Low‐/intermediate‐ risk MDS with a 5q deletion support a trend toward decreased relative risk of AML progression in patients that became TI.^15^, ^16^

Infection is recognized as a leading cause of morbidity and mortality in MDS.^17^ Neutropenia is the main predisposing factor for infections and occurs in nearly 50% of newly diagnosed patients with MDS, including 70%–80% of patients with higher‐risk MDS, and 15%–20% of patients with lower‐risk MDS.^18^ Iron overload is also known to increase risk of infection and is common in TD patients due to chronic RBC transfusions.^19^ Physicians surveyed in the present study also estimated that the number of infection events was greater in higher‐risk and TD patients.

A further important factor contributing to morbidity and mortality is thrombocytopenia‐induced bleeding. Thrombocytopenia affects up to 65% of patients with MDS and is associated with increased bleeding.^20^ Bleeding is an attributable cause of death in 13%–24% of patients with MDS.^20^, ^21^ The degree of thrombocytopenia is included in the IPSS‐R score, with worse thrombocytopenia resulting in a higher‐risk score.^6^ In the present study, physicians estimated that the number of significant bleeding events was greater in higher‐risk and TD patients.

From an economic impact standpoint, healthcare utilization among patients with MDS is high, with patients experiencing frequent hospitalizations, outpatient visits, and incurring high costs.^22^ There is a lack of studies investigating the effect of risk and TS on the number of hospitalizations. In the present study, physicians estimated that higher‐risk TD patients would experience the most hospitalizations and ER visits.

Some limitations to this study should be noted. The study is based on self‐reported data which can potentially cause such biases as inaccurate recall and false reporting. Also, data for this study was collected in April and May 2020 during the COVID‐19 pandemic; there may be possible biases in terms of physician participation across the five countries in physicians treating patients with MDS. Also, as the data is cross‐sectional in nature, assessments of causality cannot be made from the results.

## 5 CONCLUSIONS

Overall, physicians surveyed in this study estimated a lower risk of death, progression to AML, and leukemic death, fewer infections and significant bleeding events, and decreased hospitalization and ER visits for patients with MDS who are TI versus TD. Based on the physicians' clinical experience and knowledge of the literature, new treatment options for patients with MDS to reduce or eliminate transfusion burden are warranted.

## AUTHOR CONTRIBUTIONS

*Conception/design, Data Acquisition, Data Analysis, Data Interpretation*, S.G., H.C., J.G.; *Conception/design, Data Interpretation*, A.G.K., D.T.; *Data Interpretation*, J.T., S.J.

## FUNDING INFORMATION

The study was sponsored by Bristol Myers Squibb.

## CONFLICT OF INTEREST

Shaloo Gupta, Halley Costantino, and Jay Grisolano are employees of Cerner Enviza (previously Kantar Health) and have been consultants for Bristol Myers Squibb. Austin G. Kulasekararaj and Jackson Tang have been consultants for Bristol Myers Squibb. Shalon Jones and Derek Tang are employees of Bristol Myers Squibb and own stock in the company.

## ETHICS STATEMENT

The study protocol was approved with exemption status by Pearl Institutional Review Board (Indianapolis, IN, USA).

## Supporting information

Supporting Information.

## Data Availability

Due to ethical restrictions, the data for this study is stored in a secure database (in accordance with their Institution's policy on data storage); there is no public repository. However, the authors will be able to make the dataset available using secure file transfer systems (FTS) to interested parties upon request.
