# Supplementary material for: Association between transfusion status and clinical and economic outcomes in patients with myelodysplastic syndromes from the physicians' perspective
Source: Cancer Rep (Hoboken). 2022 Aug 9;6(1):e1680. doi: 10.1002/cnr2.1680 (PMC9875619; doi:10.1002/cnr2.1680)
Supplement: Supplementary file 1 — Supporting Information. [file CNR2-6-e1680-s001.docx]

**Supporting Information**

**MAIN SURVEY INTRODUCTION**

The main goal of this survey is to understand your perspectives on **the relationship between transfusion dependence** and **key clinical and economic outcomes** in Myelodysplastic Syndrome.

You will be asked to assess the **decrease in risk or chance of certain clinical outcomes** after treatment among:

- **Transfusion-independent** (TI) patients vs. **transfusion-dependent** (TD) patients (determined at baseline prior to treatment)
- **TD patients with a low transfusion burden** vs. **those with high transfusion burden** (determined at baseline prior to treatment)
- **TD patients who become TI** vs. **those who remained TD** after treatment

You will be asked to provide your perspectives for these groups. **Please provide your best estimate and evaluate each risk group individually.** The percentages do NOT need to add up to 100%.

- All MDS patients (regardless of risk levels)
- Higher-risk patients
- Lower-risk patients

Please use the following definitions when answering the questions throughout the survey:

- **Transfusion dependency**
  - Transfusion-independent (TI): <1 unit every 8 weeks
  - Transfusion-dependent (TD): ≥1 unit(s) every 8 weeks
- **Transfusion burden among transfusion dependent (TD) patients**
  - TD low burden: <4 units every 4 weeks
  - TD high burden: ≥4 units every 4 weeks
- **Risk levels**
  - Higher-risk patients
    - WHO MDS subgroups with excess blasts
    - IPSS-R scores of “Very High”, “High” or “Intermediate-2”
    - IPSS scores of “High” or “Intermediate-2”
  - Lower-risk patients
    - WHO MDS subgroups without excess blasts
    - IPSS-R scores of “Very Low”, “Low” or “Intermediate-1”
    - IPSS scores of “Low” or “Intermediate-1”

**SECTION A: CLINICAL OUTCOMES**

**Section 1:** We will explore the relationship between **transfusion dependency** and **clinical outcomes** in this section. The questions ask for your estimation of **the decrease in risk or chance of these clinical outcomes among transfusion-independent (TI) patients vs. transfusion-dependent (TD) patients.**

A1. **Decrease in Risk of Death – TI vs. TD**

What do **you** believe is the **decrease in the risk of death** among transfusion-independent (TI) patients as compared with transfusion-dependent (TD) patients for each of these groups?

| **RANGE 0–100%** | 1. MDS patients of **all risk levels** | 1. **Higher-risk** MDS patients | 1. **Lower-risk** MDS patients |
| --- | --- | --- | --- |
| **Decrease in risk of death:**  Transfusion independent **(TI) vs.** Transfusion dependent **(TD)** | __% | __% | __% |

A2. **Decrease in Risk of Death – Low vs. High Transfusion Burden Among TD Patients**

What do **you** believe is the **decrease in the risk of death** among **TD** **patients with a low transfusion burden** as compared with TD patients with a **high transfusion burden** for each of these groups?

| **RANGE 0–100%** | 1. MDS patients of **all risk levels** | 1. **Higher-risk** MDS patients | 1. **Lower-risk** MDS patients |
| --- | --- | --- | --- |
| **Decrease in the risk of death:**  **Low vs. high transfusion burden** among **transfusion-dependent (TD) patients** | __% | __% | __% |

A3. **Decrease in Risk of Death – TD Patients Who Became TI After Treatment vs. Those Who Remained TD After Treatment**

What do **you** believe is the **decrease in the risk of death** among **transfusion-dependent** **(TD) patients who became transfusion independent (TI)** after treatment, as compared with TD patients who **remained TD** after treatment across all risk levels?

_ _ _% **(RANGE 0–100%)**

A4. **Decrease in Risk of Acute Myeloid Leukemia (AML) Progression – TI vs. TD**

What do **you** believe is the **decrease in the risk of AML progression** in transfusion-independent (TI) patients vs. transfusion-dependent (TD) patients **overall across all risk levels**?

_ _ _% **(RANGE 0–100%)**

A5. **Decrease in Risk of Acute Myeloid Leukemia (AML) Progression or Death – TD Patients Who Became TI After Treatment vs. Those Who Remained TD After Treatment**

What do **you** believe is the **decrease in the risk of AML progression or death** among patients who became transfusion independent (TI) after treatment as compared with those who remained transfusion dependent (TD) after treatment for each of the following groups?

| **RANGE 0–100%** | 1. **Higher-risk** MDS patients | 1. **Lower-risk** MDS patients |
| --- | --- | --- |
| **Decrease in risk of AML progression or death**:  Patients who **became transfusion independent (TI)** after treatment **vs.** those who **remained transfusion dependent (TD)** after treatment | __% | __% |

A6. **Decrease in Risk of Non-Leukemic Death – TI vs. TD**

What do **you** believe is the **decrease in the risk of non-leukemic** **death** among transfusion-independent (TI) patients as compared with transfusion-dependent (TD) patients for each of these groups?

| **RANGE 0–100%** | 1. MDS patients of **all risk levels** | 1. **Higher-risk** MDS patients | 1. **Lower-risk** MDS patients |
| --- | --- | --- | --- |
| **Decrease in the risk of non-leukemic death:**  Transfusion independent **(TI) vs.** transfusion dependent **(TD)** | __% | __% | __% |

A7. **Decrease in Chance of Leukemia-Free Survival - TD Patients Who Remained TD After Treatment vs TD Patients Who Became TI After Treatment**

What do **you** believe is the **decrease in the chance of leukemia-free survival** among transfusion-dependent (TD) patients who remained transfusion dependent after treatment, as compared with patients who became transfusion independent (TI) after treatment for each of these groups?

| **RANGE 0–100%** | 1. MDS patients of **all risk levels** | 1. **Higher-risk** MDS patients | 1. **Lower-risk** MDS patients |
| --- | --- | --- | --- |
| **Decrease in the chance of leukemia-free survival:**  **TD patients who remained TD** after treatment vs. **those who became TI** after treatment | __% | __% | __% |

**Section 3: Number of events.**

**The questions ask for *your* estimation of occurrences of certain clinical outcomes among TI and TD patients for different risk groups.**

A10. **Infection and Significant Bleeding – Higher-Risk Patients**

What do **you** believe is the **average number** **of infection events per person per year** among transfusion-independent (TI) and transfusion-dependent (TD) MDS patients for each risk group?

Infections include sepsis, bacteremia, cellulitis, pneumonia, urinary tract/bladder infection, cystitis, sinusitis, meningitis, myocarditis, colitis, otitis media, enteritis, malaria, fungemia, thrombophlebitis, influenza, parainfluenza, HIV, West Nile virus.

|  | | **Average Number of Infection Events Per Person Per Year** |
| --- | --- | --- |
| 1 | Higher-risk, transfusion-independent (TI) patients | __________ |
| 2 | Higher-risk, transfusion-dependent (TD) patients | __________ |
| 3 | Lower-risk, transfusion-independent (TI) patients | __________ |
| 4 | Lower-risk, transfusion-dependent (TD) patients | __________ |

A11. **Significant Bleeding Events – TI vs. TD**

What do **you** believe is the **average number of significant bleeding events (GI, intracranial, hospitalized bleeding, bleeding deaths)** **per person per year** among transfusion-independent (TI) and transfusion-dependent (TD) MDS patients for each risk group?

|  | | **Average Number of Significant Bleeding Events Per Person Per Year** |
| --- | --- | --- |
| 1 | Higher-risk, transfusion-independent (TI) patients | __________ |
| 2 | Higher-risk, transfusion-dependent (TD) patients | __________ |
| 3 | Lower-risk, transfusion-independent (TI) patients | __________ |
| 4 | Lower-risk, transfusion-dependent (TD) patients | __________ |

**SECTION B: ECONOMIC OUTCOMES**

**The next questions are about the impact of transfusion dependency on the use of healthcare resources.**

B1. **Healthcare Resource Use:** **Hospitalizations**

What do **you** believe is the **average number** **of hospitalizations for any cause per person per year** in transfusion-independent (TI) and transfusion-dependent (TD) MDS patients?

|  | | **Average Number of Hospitalizations for Any Cause Per Person Per Year** |
| --- | --- | --- |
| 1 | Transfusion-independent (TI) MDS patients | __________ |
| 2 | Transfusion-dependent (TD) MDS patients | __________ |

B2. **Healthcare Resource Use:** **Emergency Room Visits**

What do **you** believe is the **average number** **of emergency room visits for any cause per person per year** in transfusion-independent (TI) and transfusion-dependent (TD) MDS patients?

|  | | **Average Number of Emergency Room Visits for Any Cause Per Person Per Year** |
| --- | --- | --- |
| 1 | Transfusion-independent (TI) MDS patients | __________ |
| 2 | Transfusion-dependent (TD) MDS patients | __________ |

**SECTION C: CLASSIFICATION**

E1. Which of the following best describes the location where you practice?

| **SELECT ONE** | |
| --- | --- |
| 1 | Urban |
| 2 | Suburban |
| 3 | Rural |

E2. How many oncologists or hematologists are in your practice, including yourself?

| **SELECT ONE** | |
| --- | --- |
| 1 | 1 |
| 2 | 2–4 |
| 3 | 5–10 |
| 4 | >10 |

E3. In which of the following age groups do you belong?

| **SELECT ONE** | |
| --- | --- |
| 1 | Under 35 years |
| 2 | 35 to 44 years |
| 3 | 45 to 54 years |
| 4 | 55 to 64 years |
| 5 | 65 years or older |

E4. Please indicate your gender.

| **SELECT ONE** | |
| --- | --- |
| 1 | Male |
| 2 | Female |
| 3 | Non-binary/third gender/transgender |
